# Ocular Albinism, Telecanthus, and Skin Depigmentation: A Phenotypic Conundrum

**DOI:** 10.7759/cureus.67204

**Published:** 2024-08-19

**Authors:** José J López-Fontanet, Raúl Y Ramos-Sánchez, Natalio Izquierdo

**Affiliations:** 1 Department of Ophthalmology, University of Puerto Rico, Medical Sciences Campus, San Juan, PRI; 2 Department of Otolaryngology-Head and Neck Surgery, University of Puerto Rico, Medical Sciences Campus, San Juan, PRI; 3 Department of Surgery, University of Puerto Rico, Medical Sciences Campus, San Juan, PRI

**Keywords:** iris transillumination, nystagmus, skin depigmentation, telecanthus, ocular albinism

## Abstract

This case report details the clinical manifestations observed in a 22-year-old male diagnosed with ocular albinism (OA). The patient underwent a comprehensive eye examination by one of the authors, revealing clinical features such as skin depigmentation, telecanthus, iris transillumination, nystagmus, and foveal hypoplasia. This report underscores the importance of a thorough clinical examination and genetic testing for accurate diagnosis, effective management, and appropriate counseling of patients with OA.

## Introduction

Ocular albinism (OA) is a hereditary eye disease characterized by a lack of pigment in the eyes, leading to various visual abnormalities [1]. Common ocular manifestations include nystagmus, photophobia, low visual acuity, iris transillumination (91% of the patients), and foveal hypoplasia [2-4]. Systemic manifestations may include hypopigmentation of the skin and hair.

The incidence of OA varies among different populations, but it is a rare condition affecting approximately 1 in 60,000 individuals [1]. OA is typically inherited as an X-linked recessive trait, with the most common mutation being in the *GPR143* gene [1,5]. Mutations in the *GPR143* gene lead to the development of oversized melanosomes with decreased motility and reduced quantity within melanocytes and retinal pigment epithelial cells [6,7]. Diagnosis is primarily based on clinical features. However, genetic testing confirms the clinical diagnosis and identifies a specific mutation leading to OA. We report on a patient with OA, wide intercanthal distance, foveal hypoplasia, and areas of skin hypopigmented macules.

## Case presentation

A 22-year-old male patient with a history of low visual acuity and nystagmus since childhood underwent a comprehensive ophthalmic evaluation by one of the authors (N.I.E.). The patient reported no medical history; his family history, including his maternal uncles and grandparents, revealed no abnormalities or pertinent ocular or dermatologic conditions.

**Figure 1:**
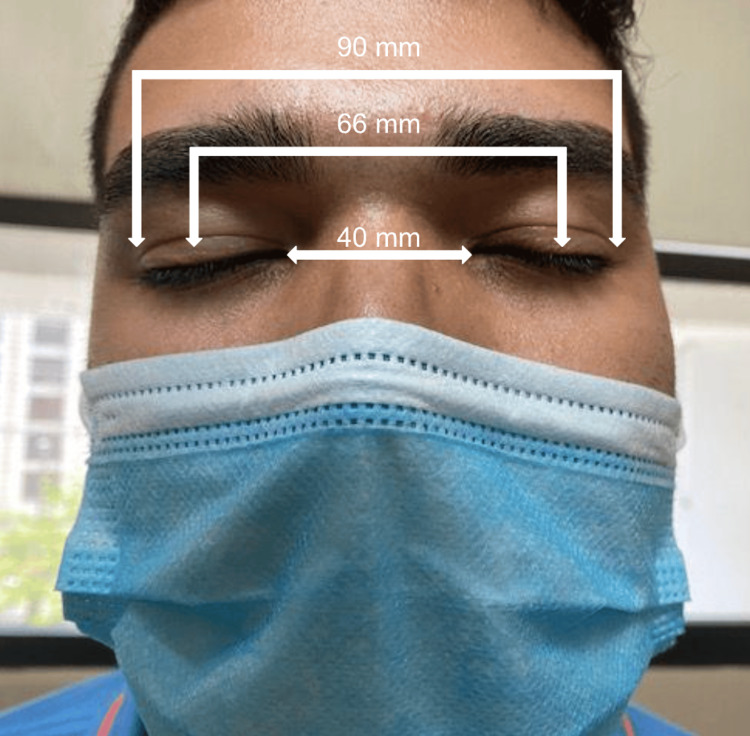
A frontal view of the patient's face, illustrating the measurements of the intercanthal distance, interpupillary distance, and outer canthal distance. To respect the patient's privacy, the interpupillary distance is marked with the patient's eyes closed, indicating the location of the pupils

The best corrected visual acuity was 0.7 and 0.6 in the right (oculus dextrus, OD) and left eye (oculus sinister, OS), respectively (logarithm of the minimum angle of resolution). Refraction was -1.75 + 3.50 × 90° and -3.00 + 2.50 × 90° for the OD and OS, respectively. As shown in Figure 1, the intercanthal distance measured 40 mm, the interpupillary distance was 66 mm, and the outer canthal distance was 90 mm, with a W index of 2.14, confirming telecanthus.

**Figure 2:**
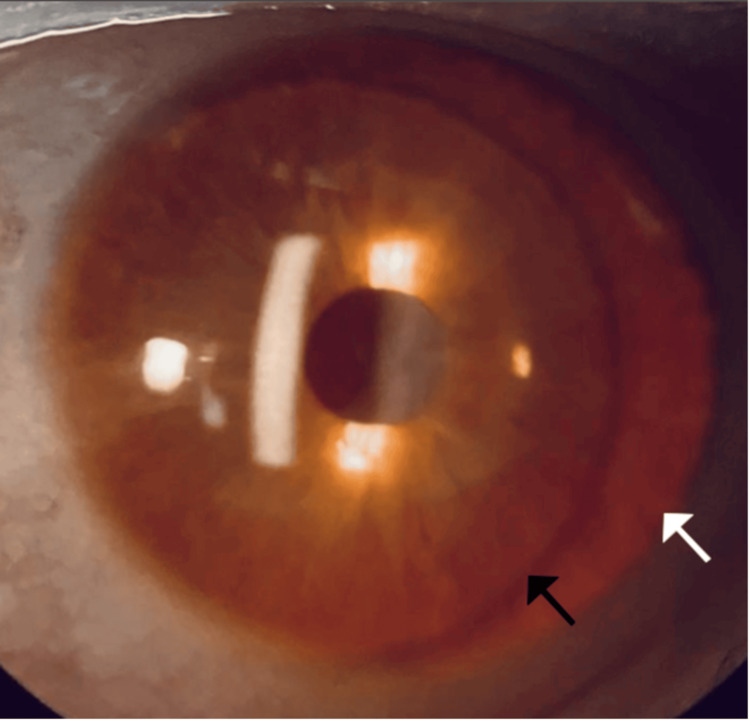
Peripheral iris transillumination. The black arrow demarcates the intraocular lens, while the white arrow demarcates the peripheral transillumination

**Figure 3:**
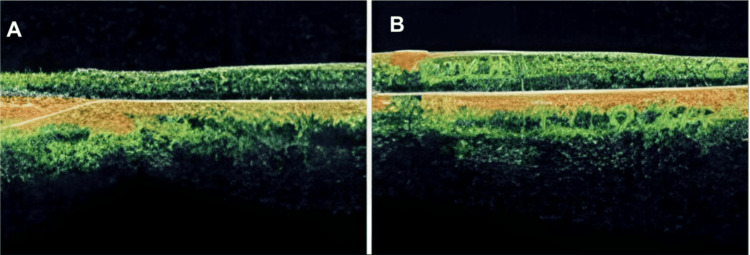
Optical coherence tomography examination of the macula. The macula of the (A) right and (B) left eyes shows loss of the foveal pit with grade 3 foveal hypoplasia

Upon slit lamp examination, as depicted in Figure 2, the patient had peripheral iris transillumination. Fundus examination revealed normal optic nerves and vessels. Additionally, there was a lack of foveal and macular pigmentation, with the presence of albinotic mid-peripheries. The patient's macular optical coherence tomography (Carl Zeiss Meditec, Inc., Dublin, CA) shows foveal hypoplasia, classified as grade 3 based on morphologic features, with a 207-micron OD and 236-micron OS thickness. The total macular volume measured 5.67 mm³ OD and 6.26 mm³ OS, as depicted in Figure 3.

Upon visual field evaluation using the 30-2 protocol by Humphrey Field Analyzer 3 (Carl Zeiss Meditec, Inc.), the patient had a statistically significant mean deviation of -9.26 dB (p < 0.5%) OD and -3.89 dB (p < 1%) OS, along with a pattern standard deviation of +6.61 dB (p < 0.5%) OD and +4.30 dB (p < 0.5%) OS.

**Figure 4:**
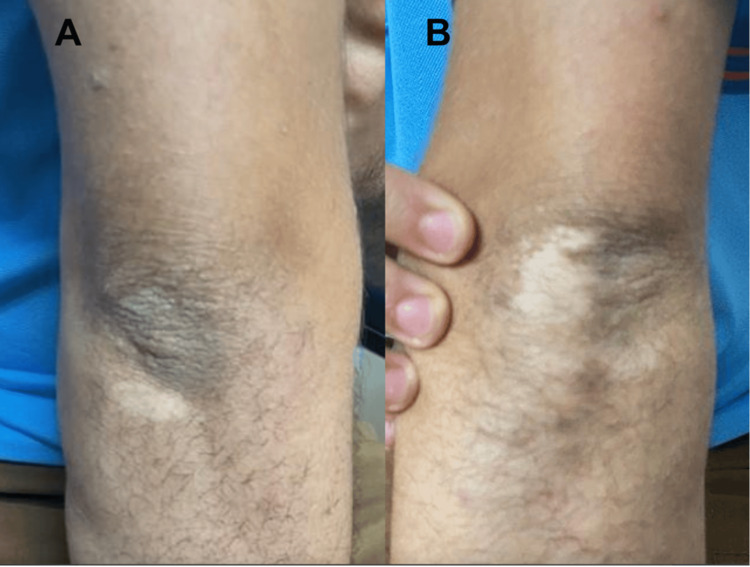
(A,B) A visual depiction of the skin patchy depigmentation observed in the posterior aspect of both elbows of the patient

**Figure 5:**
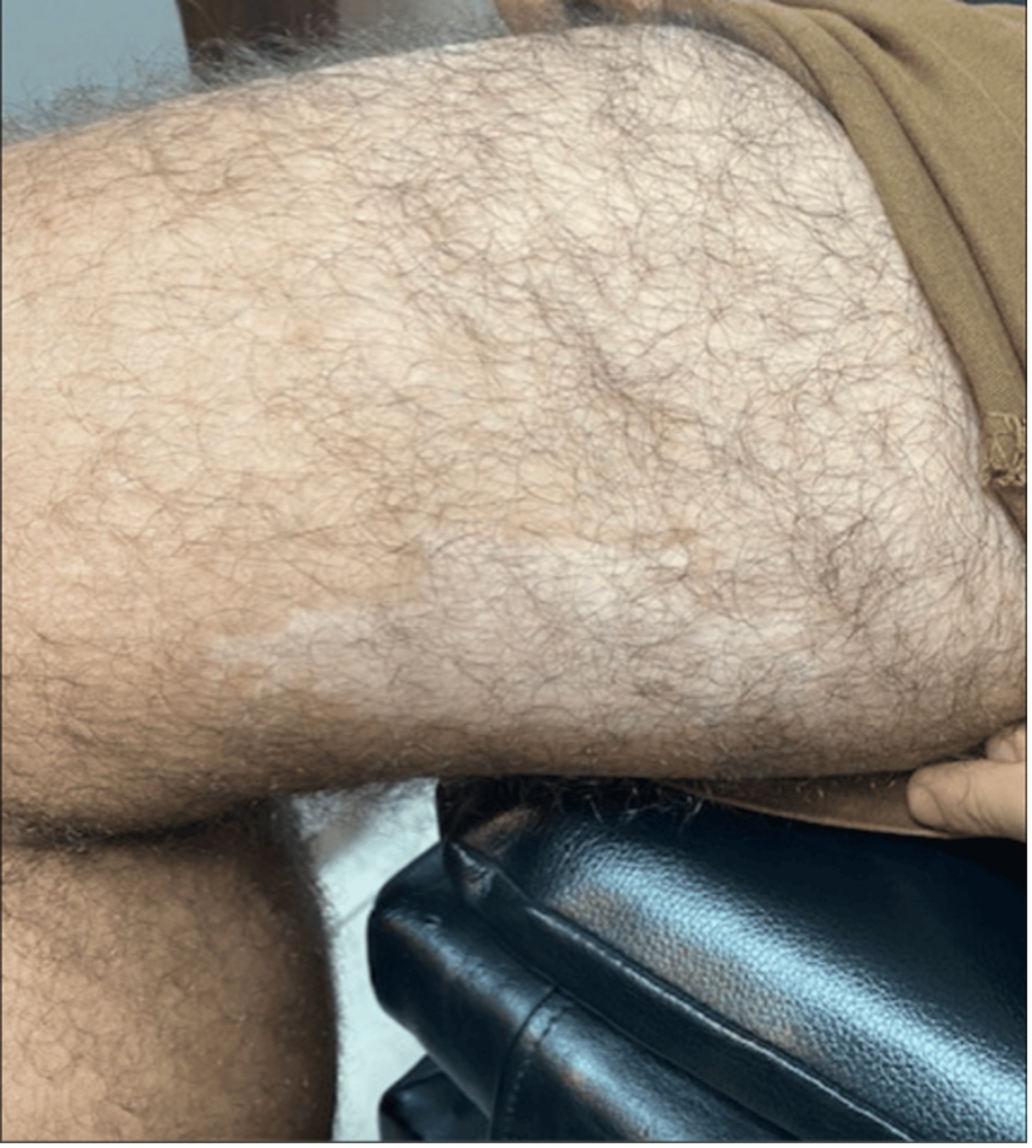
A visual depiction of the skin patchy depigmentation observed in the medial thigh of the patient

Upon systemic physical examination, the patient had skin patchy depigmentation in the posterior aspect of both elbows and the medial area of the right thigh, as shown in Figures 4, 5. The patient was referred to a dermatologist for a differential diagnosis of hypopigmented skin lesions, including Waardenburg syndrome, vitiligo, and piebaldism. Vitiligo was ruled out with histopathologic studies.

Audiologic testing showed bilateral normal hearing. Impedance audiometry results indicated normal middle ear function in both ears, confirmed by consistent otoacoustic emissions.

Next-generation gene sequencing and deletion/duplication analysis were performed using the Invitae Corporation (San Francisco, CA) gene panel for dermatologic diseases. The results showed a hemizygous missense mutation in gene *GPR143* c.905A>G (p.Gln302Arg), reported as a variant of uncertain significance.

## Discussion

OA is a hereditary disease primarily affecting the eyes, with common manifestations such as nystagmus, iris transillumination, and foveal hypoplasia. It is typically inherited in an X-linked recessive manner, with the *GPR143* gene being the most frequently implicated gene. Our patient had poor vision, nystagmus, iris transillumination, and foveal hypoplasia. This was confirmed with optical coherence tomography that showed loss of the foveal pit and reduced foveal thickness, as depicted in Figure 3. These findings correlate with the literature on OA.

Previous studies have reported on patients with Waardenburg syndrome as having hypopigmented macules and telecanthus, hearing loss, and changes in hair, skin, and pigmentary iris changes [8], which can sometimes present with symptoms overlapping with OA. Our patient did not have audiology problems but had an increased intercanthal distance, measuring 40 mm. This feature is not a primary characteristic of OA. To our knowledge, this is the first report of telecanthus in a patient with OA.

Vitiligo is an autoimmune disease that leads to skin depigmentation [9]. Our patient presented with hypopigmented macules on both elbows and the right thigh, which raised the suspicion of vitiligo as part of the differential diagnosis. However, after further evaluation by a dermatologist, vitiligo was excluded in this patient.

Previous studies have reported on the ocular findings of patients with OA due to the X-linked *GPR143* gene mutations [1]. Our patient’s findings are compatible with the literature. However, genetic testing proved pivotal in reaching this patient's diagnosis. To our knowledge, this is the first report of this gene variant (c.905A>G (p.Gln302Arg)) leading to OA.

## Conclusions

Patients with OA require a comprehensive ophthalmic and physical examination. In this case, the patient had phenotypic variations that can be associated with other diseases, highlighting the importance of a thorough clinical evaluation coupled with genetic testing, which accurately diagnoses and manages patients with OA.

While genetic testing has become an increasingly valuable tool for diagnosing and managing OA patients, it is essential to acknowledge its technological limitations. Challenges such as variants of unknown significance, incomplete testing, and unidentified related genes should be considered when interpreting genetic findings.
